# Successful treatment of refractory chilblain lupus erythematosus with bosentan

**DOI:** 10.1093/rheumatology/keaf444

**Published:** 2025-08-13

**Authors:** Shannon Gunawardana, Alexander J Clarke, Antonia Lloyd-Lavery, Shirish Dubey

**Affiliations:** Department of Rheumatology, Oxford University Hospitals NHS FT, Oxford, United Kingdom; Kennedy Institute of Rheumatology, University of Oxford, Oxford, United Kingdom; Department of Dermatology, Oxford University Hospitals NHS FT, Oxford, United Kingdom; Department of Rheumatology, Oxford University Hospitals NHS FT, Oxford, United Kingdom

Rheumatology key message

- Bosentan may be a useful treatment option for secondary chilblains refractory to conventional treatments.

Dear editor, A 39-year-old female with a background of Crohn’s disease presented to the Dermatology team in 2021 with chilblains affecting the feet, particularly during the cooler months. She described her toes becoming erythematous and oedematous with an associated paraesthesia; subsequently, there would be distal purple discolouration with occasional ulceration. There were typical triphasic colour changes of the distal extremities consistent with RP. She was an active smoker of 20 cigarettes daily. She had occasional mouth ulcers that could be attributable to Crohn’s disease, and no other symptoms of CTD. ANA and cryoglobulin screening were negative, and her lower limbs arterial duplex scan was normal. A recommendation to keep her peripheries warm and smoking cessation advice was given, and treatment commenced with nifedipine 5 mg three times daily.

The patient re-presented to the Dermatology emergency clinic in May 2023 with acute severe pain in the left toes following unsuccessful treatment with flucloxacillin in primary care. She underwent a four-millimetre punch biopsy from the left toe. This was performed cautiously given the risks associated with performing a skin biopsy in areas with poor perfusion, where it can trigger tissue necrosis or non-healing. She was prescribed a 3-week course of clobetasol propionate ointment and potassium permanganate soaks to treat the areas of ulceration. ANA was now weakly positive with a speckled pattern (1:160) with negative dsDNA and ENA panel (including Scl-70 antibody). aPL screening and ANCA were negative; protein electrophoresis, immunoglobulins and complement C3 and C4 levels were normal.

Histopathology of the punch biopsy showed slightly disrupted dermis, superficial and deep peri-adnexal, perivascular lymphocytic infiltrate, and associated mild dermal papillae oedema. A subtle interface reaction and mucin deposition were seen, suggestive of chilblain lupus erythematosus (CHLE).

Given the unusual presentation, a broad differential diagnosis including SSc, DM, Buerger’s disease, and Behçet’s disease were considered. However, there were no supporting features to support a diagnosis of any of these conditions, although the patient may develop other features in due course.

On review in the combined Dermatology–Rheumatology clinic in May 2023, HCQ 200 mg twice daily was commenced; nifedipine and topical treatments were continued. Despite this regime, the patient had ongoing RP of the toes on the left foot. Nifedipine was switched to sildenafil 25 mg three times daily. The patient had stopped smoking.

In December 2023, the patient reported ongoing severe chilblains, associated with painful ulceration that was limiting her ability to work. Pharmacological options, including immunosuppression, were discussed, and a decision was made to start bosentan 62.5 mg twice daily, increasing to 125 mg twice daily after 2 weeks. The patient had an excellent response to this. She was most recently reviewed in January 2025 and continues to take HCQ 200 mg twice daily, sildenafil 50 mg three times daily and bosentan 125 mg twice daily. She reports that the addition of bosentan has made the most significant difference to improving her symptoms by reducing the frequency and severity of chilblain flares. Nailfold capillaroscopy, performed after bosentan was commenced, did not demonstrate any giant capillaries, increased tortuosity or dropouts to suggest SSc or active CTD (Fig. 1).

**Figure 1.**
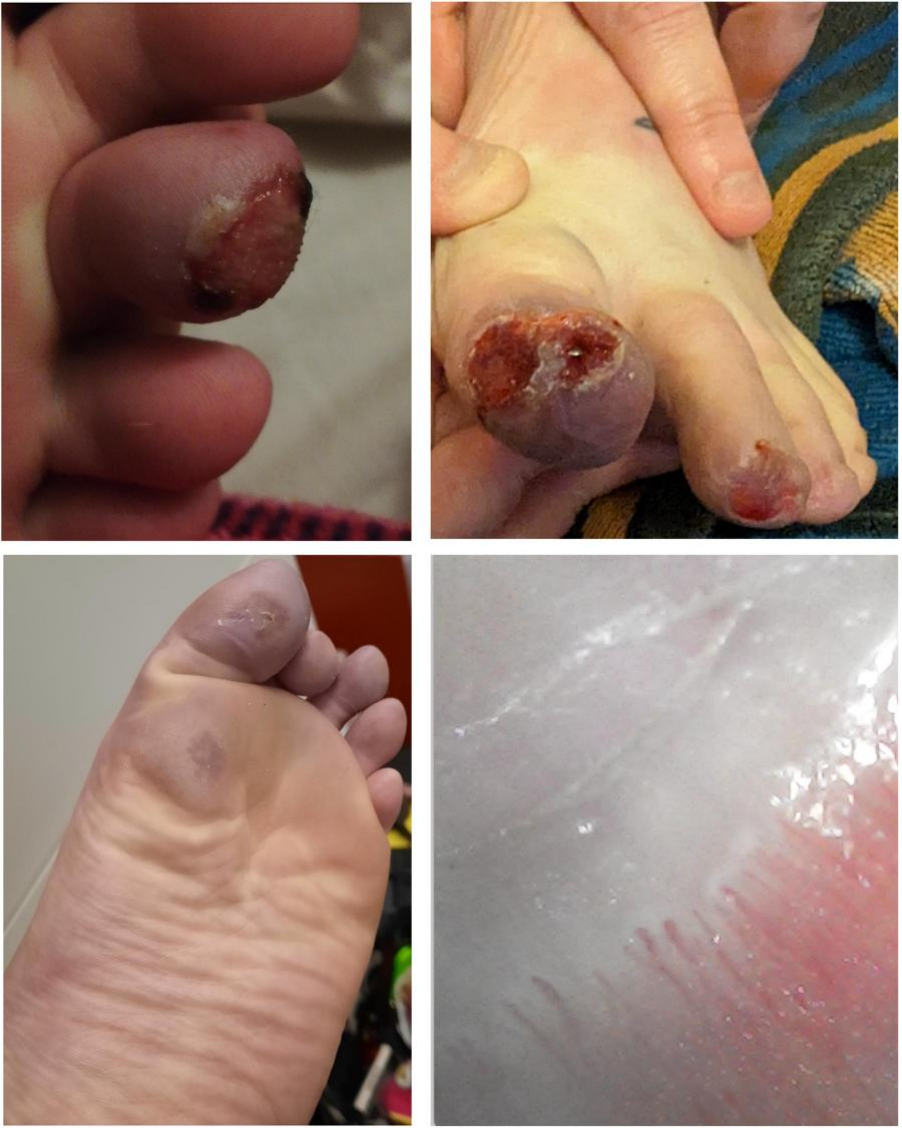
Chilblain lesions affecting the patient’s left foot, and nailfold capillaroscopy of the left middle finger

This patient has so far been followed up for 3 years and has not yet evolved any features that would support an alternative diagnosis to CHLE.

Chilblains, or pernioses, are inflammatory cutaneous lesions in which the extremities become painful, erythematous, cyanotic, pruritic and oedematous in response to cold or damp conditions [1]. Chilblains can be idiopathic or secondary, associated with immune-mediated inflammatory disorders, infection, haematological disorders, malignancy or drug-reactions. The most common form of secondary chilblains is chilblain lupus erythematosus (CHLE) [2]. Idiopathic chilblains and sporadic CHLE most commonly affect middle-aged women and affect the toes more frequently than the fingers [3].

The histological and immunopathological differences between idiopathic chilblains and CHLE have previously been described [1]. Wang *et al.* reported a comparative analysis of histopathological features of skin biopsies and proposed interstitial fibrin and dermal mucin as features supporting a diagnosis of CHLE [4]. The pathogenesis of CHLE is not fully understood. Current understanding is that cold weather provokes vasoconstriction or microvascular trauma that causes occlusion of the capillary bed with aggregated red blood cells contributing to hyperviscosity [1, 2].

Conservative measures of avoiding cold and damp conditions by physical barriers and warming is broadly accepted in the management of chilblains and CHLE. There is some evidence for the use of drugs such as nifedipine, pentoxifylline and tadalafil in the treatment of primary chilblains, but there are no trials investigating the management of CHLE [1]. The therapeutic armamentarium is rather limited, and newer therapies are needed. This is the first report describing improvement of chilblains (CHLE) with bosentan in a patient with refractory symptoms.

Endothelin-1 is a potent vasoconstrictor that acts through endothelin-A and endothelin-B receptors [5]. Bosentan, a dual endothelin-receptor, is licensed for the treatment of digital ulcers in SSc [6]. The RAPIDS-2 double-blind placebo-controlled trial demonstrated that bosentan reduced the occurrence of new digital ulcers by ∼30% in patients with SSc [7]. In a small study of 18 patients, endothelin-1 concentrations were increased at baseline and following cold exposure in patients with RP and essential acrocyanosis, compared with healthy controls [8]. This suggests that endothelin-1 might contribute to the abnormal vasoconstriction response in acrocyanotic conditions. The vasodilatory effects of bosentan could explain its efficacy in chilblains. Historically, bosentan has been a high-cost drug, which has limited its use [6].

The endothelin pathway has not been investigated in the pathophysiology of chilblains, and this case suggests there would be value in doing so. Bosentan might provide important benefit for patients with refractory chilblains and warrants further investigation in randomized controlled trials in CHLE.

Informed patient consent was provided for the publication of this article.

## Data Availability

Data are available upon request.
